# Using shared needles for subcutaneous inoculation can transmit bluetongue virus mechanically between ruminant hosts

**DOI:** 10.1038/srep20627

**Published:** 2016-02-08

**Authors:** Karin E. Darpel, James Barber, Andrew Hope, Anthony J. Wilson, Simon Gubbins, Mark Henstock, Lorraine Frost, Carrie Batten, Eva Veronesi, Katy Moffat, Simon Carpenter, Chris Oura, Philip S. Mellor, Peter P. C. Mertens

**Affiliations:** 1 The Pirbright Institute, Woking, Surrey, United Kingdom; 2 School of Veterinary Medicine, University of Surrey, Guildford, United Kingdom; 3 Vector Biology Department, Liverpool School of Tropical Medicine, Liverpool, United Kingdom; 4 Institute of Parasitology, University of Zurich, Zurich, Switzerland; 5 School of Veterinary Medicine, University of the West Indies, Trinidad and Tobago

## Abstract

Bluetongue virus (BTV) is an economically important arbovirus of ruminants that is transmitted by *Culicoides spp*. biting midges. BTV infection of ruminants results in a high viraemia, suggesting that repeated sharing of needles between animals could result in its iatrogenic transmission. Studies defining the risk of iatrogenic transmission of blood-borne pathogens by less invasive routes, such as subcutaneous or intradermal inoculations are rare, even though the sharing of needles is common practice for these inoculation routes in the veterinary sector. Here we demonstrate that BTV can be transmitted by needle sharing during subcutaneous inoculation, despite the absence of visible blood contamination of the needles. The incubation period, measured from sharing of needles, to detection of BTV in the recipient sheep or cattle, was substantially longer than has previously been reported after experimental infection of ruminants by either direct inoculation of virus, or through blood feeding by infected *Culicoides*. Although such mechanical transmission is most likely rare under field condition, these results are likely to influence future advice given in relation to sharing needles during veterinary vaccination campaigns and will also be of interest for the public health sector considering the risk of pathogen transmission during subcutaneous inoculations with re-used needles.

The risk of iatrogenic transmission of pathogens through blood-contaminated needles and surgical equipment is widely recognised in both veterinary and human medicine^1^,^2^,^3^,^4^. Studies published on this subject usually report iatrogenic transmission of pathogens in humans and animals either through a ‘blood-to-blood’ transmission route (e.g. by transfusion, contaminated surgical equipment or the sharing of needles for intravenous or intramuscular inoculation), or a blood to broken skin/mucosal route (e.g. by blood splashes or dehorning equipment)^5^,^6^.

Although iatrogenic transmission of important veterinary viruses such as bovine leukaemia virus (BLV) , bovine viral diarrhoea virus (BVDV), equine infectious anaemia virus (EIAV) and porcine reproductive and respiratory syndrome virus (PRRSV) has been reported, transmission in these studies was associated with much more invasive techniques such as dehorning^5^,^7^,^8^, ear tattooing^9^, minor surgery or the re-use of hypodermic needles by intravenous or intramuscular routes^1^,^3^,^10^. Experiments that define the risk of transmission through subcutaneous and intradermal inoculation routes have been only rarely conducted^11^,^12^ or did not result in successful transmission or higher transmission risks^13^,^14^,^15^.

Subcutaneous or intradermal inoculations do not usually result in visible contamination of the needle with blood and the risk of transmission of blood-borne pathogens may be assumed to be low^13^,^15^,^16^. Veterinary and agriculture workers frequently re-use needles when inoculating animals via these routes and it is therefore important that the pathogen-transmission risks for these procedures are adequately assessed. For example, routine mass vaccination of livestock is often carried out using automatic syringes or vaccination guns, which may re-use the same needles between individuals (although some devices do superficially clean the needle between injections) (http://academy.fwi.co.uk/Courses/Livestock/Diseases-in-cattle/Bluetongue).

Alternative transmission routes need to be taken into consideration when assessing the overall risk of the incursion and spread of viruses^17^,^18^,^19^. This is particularly important for those viruses which are not highly contagious by direct contact, but are transmitted very effectively by other specific routes, such as arthropod vector bite or sexual transmission^20^,^21^. Examples of viruses that are spread through blood-contaminated needles, particularly in high risk groups (such as intravenous drug users or healthcare workers) include Hepatitis B, C and HIV^21^. In humans, viral transmission through needle-prick injuries or mucocutaneous blood contact has also been reported for several arthropod-borne viruses (arboviruses), including dengue fever virus^11^,^22^.

Bluetongue virus (BTV), an economically important arbovirus, is primarily biologically transmitted between its ruminant hosts by *Culicoides* biting midges^23^. In the last decade BTV has dramatically expanded its distribution range into Europe, causing severe disease in northern European sheep, as well as high morbidity levels in cattle^24^,^25^,^26^. During the mass vaccination campaigns employed to control these outbreaks^27^, concerns were raised about the potential for BTV to be transmitted by the use of shared needles, during sequential subcutaneous inoculations.

In this study we have investigated the potential of BTV to be mechanically transmitted from infected donor to non-infected recipient ruminants when carrying out minimally invasive subcutaneous or intradermal injections while re-using the same needle in the absence of visible blood contamination. These results are likely to influence future advice given to veterinary policymakers, farmers and veterinarians in relation to sharing needles during vaccination campaigns and will also be of interest to human health officials when considering the risk of pathogen transmission during subcutaneous inoculations with re-used needles.

## Results

### Infection of donor animals

**Figure 1:**
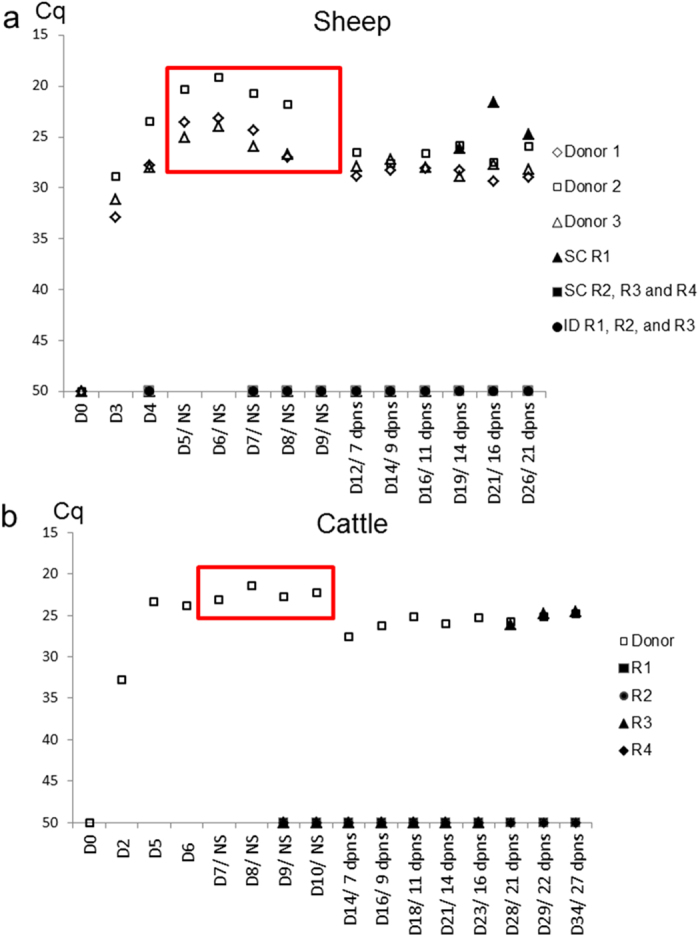
BTV RNA detection by rRT-PCR in blood of donor and recipient ruminants. Panel (**a**) BTV RNA was detected in all donor sheep (inoculated subcutaneously with 1 ml tissue culture supernatant (TCS) containing 10^7^ TCID_50/_ml of BTV-8KC2) by 3 days post infection (representing the first sampling day). On days marked with NS, either subcutaneous or intradermal inoculations were carried out using a shared needle between donors and recipients. These days (D5-D9 post infection) correlated with the days of highest donor RNAaemia (red box). BTV RNA was detected in recipient SC1 at 14 days post needle sharing (dpns) as counted from the first needle sharing attempt. All other recipient sheep, subcutaneous or intradermal, remained negative until the end of the study. Panel (**b**) The donor cattle (inoculated subcutaneously with 1 ml tissue culture supernatant (TCS) containing 10^7^ TCID_50/_ml of BTV8-KC2) had detectable level of BTV RNA in systemic blood by 2 days post infection (the first day of sample analysis). Subcutaneous inoculations by shared needle were carried out between D7-D10 post infection, again correlating with highest donor BTV RNA level (red box). Recipient 3 tested positive for BTV RNA in the blood at 21 dpns, while all other recipient cattle remained negative until the end of the study.(D1 = Day 1 post infection; NS = needle sharing; dpns = days post needle sharing).

BTV replication was confirmed in the BTV-8KC2 inoculated donor animals by rRT-PCR (Fig. 1). On the days of shared needle inoculations, the donor animals (sheep D5-D9 post-donor infection; cattle D7–10 post-donor infection) showed peak BTV RNA detectable in the blood (i.e. the lowest C_q_-value) for all 4 donor animals (Fig. 1). The two days of highest BTV RNA levels in the blood were chosen for virus titration on KC-*C. sonorensis* cells to confirm the presence of infectious virus. Viral titres in donor animals ranged from 10^4.5^ TCID_50_/ml to 10^6.25^ TCID_50_/ml on the days tested (Table 1).

Serum samples from all donor sheep were initially tested at 12 days post-infection (dpi), by which time all donor animals had detectable levels of BTV specific immunoglobulins (D1 276% S/P; D2 213% S/P; D3 376% S/P) and remained positive until the end of the experiment on 26 dpi. The serum from the donor bull was first tested at 14 dpi, by which time the animal tested positive for BTV immunoglobulins (334% S/P) and remained positive until the end of the experiment at 34 dpi.

### Testing of Recipient animals

Only one of the subcutaneous recipient sheep (SC-R1) tested positive for BTV RNA (Fig. 1: C_q_ = 26.1; 26.1) in blood samples taken 14 days after the first subcutaneous needle sharing event (11 days following the last needle sharing event). Prior to this point, (including a blood sample taken 3 days earlier) no BTV RNA had been detected in blood samples from this sheep. A further two blood samples from the same animal also contained detectable BTV RNA (Fig. 1). Infectious virus was isolated from all three BTV RNA positive blood samples of this recipient sheep and the viral titre was determined as 10^4.75^ TCID_50_/ml in the first BTV RNA positive blood sample. On the first day on which BTV RNA and infectious virus was detected in the recipients’ blood, no BTV specific immunoglobulins were detected in the serum. However the recipient sheep was found to have seroconverted by the end of the experiment 7 days later (272% S/P).

The BTV positive recipient sheep (SC-R1) showed mild clinical signs consistent with bluetongue (BT), 4 days after initial detection of BTV RNA/infectious virus in its blood. These signs included reddening of the mucosal membrane, swollen upper and lower lips followed by reddening of the coronary bands. The peak temperature recorded was 39.9 °C, although the onset of higher fever might have been missed as the frequency of body temperature recording had been reduced at this stage of the study to coincide with sampling days. The animal was euthanized seven days after detection of BTV infection and the pathological signs recorded were typical for BTV infection and included multiple swollen and haemorrhagic lymph nodes and petechial bleeding in the oral cavity.

None of the other three subcutaneous recipient sheep, or the three intradermal recipient sheep had detectable levels of BTV-RNA in their blood (Fig. 1), BTV immunoglobulin in their serum, or exhibited clinical signs by the end of the experiment at 21 days after the first needle exchange.

Cattle recipient 2 (SC-RC2) tested positive for BTV RNA (Fig. 1: C_q_ = 25.8; 26.7) in blood samples taken 21 days following the first subcutaneous needle sharing event (18 days following the last needle sharing event). On the preceding sampling day (5 days earlier), the blood from this recipient did not contain detectable BTV RNA. Two further blood samples taken from SC-RC2 also tested positive for BTV RNA (Fig. 1) and infectious virus was isolated from each of the positive blood samples. The viral titre in the first BTV RNA positive sample was 10^3.5^ TCID_50_/ml. By the end of the experiment (7 days after the first positive BTV RNA rRT-PCR result) the animal had not yet seroconverted and no BTV specific antibodies were detected in its serum (13% S/P, classed negative as below the positive threshold of 30%). No clinical signs of BT were observed in the recipient animal throughout the experiment.

### Probability of mechanical transmission

For each of the three experiments conducted (cattle, sheep SC, sheep ID), the 95% credible interval (CI) for the estimated effect of the C_q_ value of the donor animal on the probability of transmission included zero. Consequently, there is no evidence of an effect of the C_q_ value of the donor animal on the probability of transmission and all subsequent results are presented for the model as independent of donor C_q_.

For the experiment in which transmission between cattle was attempted via subcutanous inoculation, the probability of transmission for each challenge was estimated to be 0.0243 (95% CI 0.0185–0.102), the mean incubation period to be 3.73 days (95% CI 3.14–4.25), and the shape parameter for the incubation period distribution to be 0.801 (95% CI 0.143–2.77).

Where transmission between sheep was attempted via subcutaneous inoculation, the probability of transmission for each challenge was estimated to be 0.0189 (95% CI 0.00139–0.0794), the mean incubation period to be 2.57 days (95% CI 2.15 –3.32), and the shape parameter for the incubation period distribution to be 1.33 (95% CI 0.208–5.89]).

For the experiment in which transmission between sheep was attempted via intradermal inoculation, the probability of transmission for each challenge was estimated to be less than 8.91 × 10^−10^ (95% CI 3.33 × 10^−16^–0.00175), the mean incubation period to be 2.51 days (95% CI 2.14–3.20), and the shape parameter for the incubation period distribution to be 6.09 (95% CI 1.30–10.4).

## Discussion

This study demonstrates for the first time that BTV can be transmitted between infected and uninfected ruminants through subcutaneous inoculations using the same needle. In the wider literature, iatrogenic transmission of blood-borne pathogens is normally associated with more invasive techniques such as needle prick injuries (muscle layer), intramuscular and intravenous inoculations, or surgical procedures^7^,^10^,^12^.

Subcutaneous and intradermal inoculations do not normally result in the transfer of blood and no visible blood contamination was observed on the needle in the current experiments. In several previously published epidemiological studies common vaccination practises did not increase the risk of BLV transmission^14^,^15^ and experimental transmission between infected and non-infected animals using common needle injection techniques (intramuscular or intradermal route) failed, unless the needle was specifically covered in viraemic blood^13^,^16^.

However in our study, one of the four recipient sheep and one of the four recipient cattle became infected following a sequence of multiple alternating subcutaneous inoculations from infected donor to naïve recipients, demonstrating that sharing needles, even by the minimally invasive subcutaneous route, can result in BTV transmission. We have recently highlighted that BTV can replicate directly in the endothelial cells of skin capillaries^28^, hence the presence of replicating virus in the skin might offer a possible explanation for needle contamination with virus without visible blood resulting in iatrogenic transmission of BTV during sequential subcutaneous inoculations.

In the current study, the two recipient animals which demonstrated a successful transmission event also exhibited prolonged incubation periods until BTV was detected in the blood (as confirmed by the presence of viral RNA or infectious virus), compared to other studies using early detection technologies such as real-time RT-PCR. Ruminants inoculated with BTV during *in vivo* studies mostly develop BTV RNA levels detectable by RT-PCR or real-time RT-PCR in the blood by day 1 to day 7 post-infection^29^,^30^,^31^,^32^ depending on ruminant species and breed. Although BTV incubation periods in ruminants of 2–16 days have previously been reported^33^,^34^, recent advances in virus detection technologies create difficulties when comparing historic infection studies that relied on virus isolation to those utilising RNA detection by rRT-PCR^35^, the latter consistently reporting early detection between 1–7 days post infection^35^,^36^,^37^. Furthermore the sheep breed used in this trial (Dorset Poll) has previously been reported to be highly susceptible to BTV infection and the viral RNA is normally detected in the blood between day 1 and-day 5 post infection^31^,^32^,^37^. Early BTV RNA detection was also observed in the four donor animals in this study, all of which had detectable BTV RNA in their blood by 2 or 3 days post infection.

In many viral diseases, such as foot-and-mouth disease (FMD), a higher inoculation dose of virus seems to result in shorter incubation times until viraemia can be detected^38^,^39^. Studies for BTV have revealed little change in incubation periods in relation to infectious dose, with infected animals still being reported BTV positive by day 7 post infection, even when low viral dosage were administered^40^,^41^.

In a previous study using BTV, cattle were shown to have detectable RNA and infectious virus in their blood stream by 5 dpi despite using an inoculation dose as low as 10 TCID_50_/ml^41^. Interestingly one of the five calves inoculated within a low titre infection group, failed to develop detectable viraemia (RNA or infectious virus) throughout the experiment, which tested the blood of calves for BTV RNA by rRT-PCR for >160 days^41^.

The extended time period until detection of BTV RNA by rRT-PCR in the sheep and bovine recipients in our study can therefore be regarded as unusual. However, despite the prolonged incubation periods observed, the subsequent infection kinetics (determined as levels of BTV RNA in the blood) in the two recipients animals were comparable to the directly infected donors (Fig. 1). Furthermore sheep recipient 1 developed clinical signs and pathology typical of BT by the end of the study.

Nonetheless some studies infecting sheep via the bites of infected *Culicoides* spp. have also reported longer incubation periods of 15 or 16 days duration before infection was confirmed^34^,^42^. These studies include a recent investigation using intra-thoracically (systemically) infected *C. nubeculosus,* that were allowed to blood-feed on sheep, and which reported BTV RNA detectable by rRT-PCR in 6 out of the 8 sheep between 2–7 days post *Culicoides* feeding, but only at 15 dpi in the 2 remaining sheep^42^. BTV infection of sheep using infected insects is difficult to compare with other methodologies as the infectious dose cannot be easily quantified, but it might suggest that very low infectious doses could take longer to result in systemic detection of the virus.

The long incubation period observed in the current study might influence the perceived association of the event “inoculation using shared needle” with the outcome “bluetongue virus infections”. Should ruminants be tested positive for BTV 3–4 weeks after a routine vaccination, farmers and veterinarians might assume a natural infection rather than associating infection with the inoculation practises.

The closeness of the observed incubation times in the BTV8 infected recipient animals to the endpoint of the experiments further increased uncertainty over the incubation period required to detect successful mechanical transmission by subcutaneous needle sharing. Recipients which remained negative could represent either an unsuccessful transmission or a successful transmission for which the incubation period was not yet complete thereby making it more difficult to estimate the probability of mechanical transmission by this route.

It is also important to acknowledge that each recipient animal received an unusually high number of subcutaneous or intra-dermal inoculations by shared needles, suggesting that mechanical transmission by these routes is likely to be rare under field conditions. Estimates of transmission probabilities obtained in our analysis under the assumption of comparable incubation periods between typical experimental infection and mechanical needlestick transmission also suggested the latter to represent a rare event. Some automated vaccination guns also have devices to superficially clean the needles between injections, which might further reduce the risk for iatrogenic transmission during subcutaneous inoculation in the field, however this would need to be verified by further studies.

Nonetheless this does not preclude this route of transmission playing a significant role in the epidemiology of BTV and our estimates are likely to be strongly influenced by the potential duration of the incubation period. Further studies which include follow-up of recipient animals for longer periods are needed to improve estimation of transmission efficiency via this route.

In summary this study demonstrates for the first time that BTV transmission is possible by sharing needles for subcutaneous injections and that such transmission might occur in the field, most likely at a low, but currently unknown frequency. The successful transmission of an arbovirus by a relatively non-invasive technique such as subcutaneous inoculation, in the absence of visible blood contamination, is relevant to veterinarians and farmers, as well as to the medical and public health community. In light of these results, it would be advisable to consider disposable needle use during herd vaccination campaigns carried out in areas and seasons of BTV circulation to prevent any risk of iatrogenic BTV transmission.

## Materials and Methods

### Study Materials and Animals

Bluetongue virus isolations and titrations were carried out in KC cells derived from *Culicoides sonorensis* embryos (KC-*C. sonorensis*)^43^. These were grown in Schneider’s insect cell medium (Lonza) and supplemented with 1% Penicillin/Streptomycin (Sigma), 1% Amphotericin B (Sigma) and 10% heat-inactivated foetal-calf serum (FCS) (Gibco) (‘growth media’).

The virus used as inoculum was isolated from a blood sample taken from the UK index case cow infected with BTV-8 in 2007. Washed and sonicated blood was directly added to KC-*C. sonorensis* cells in growth media. The virus was passaged a second time in KC-*C. sonorensis* cells and was designated: BTV8KC2 (Orbivirus Reference Collection number: UKG2007/64). The titre of the donor inoculum used was determined by titration on KC-*C. sonorensis* cells (see below) as 10^7^ TCID_50_/ml.

Five 9-month old male Holstein Friesian cattle and ten 6–12 month old Dorset Poll sheep of both sexes were kept in insect-proof isolation units at The Pirbright Institute.

### Ethical statement animal studies

All animal experiments were carried out in accordance with the UK Animal Scientific Procedure Act (ASPA) 1986 which transposes European Directive 2010/63/EU into UK national law.

The animal studies were approved by the UK home office in granting Project licence 70/6798 under the Animal Scientific Procedure Act and all protocols had undergone appropriate local ethical review procedures by the Animal welfare and Ethical Review Board (AWERB) of The Pirbright Institute.

### Experimental design

#### Sheep

Three sheep were assigned to a “donor” group and inoculated subcutaneously with 1 ml (10^7^ TCID_50_) BTV-8KC2 into the left side of the neck. Four sheep were assigned as “subcutaneous recipients” and 3 sheep as “intradermal recipients”.

#### Subcutaneous recipients

On day 5 post inoculation of the donor sheep a 10 ml syringe was filled with sterile PBS and, using a 20 g needle, 1 ml of PBS was inoculated subcutaneously into the neck of the donor animal according to standard veterinary practice. Immediately after, a further 1 ml of PBS was inoculated subcutaneously into the neck of a recipient sheep using the same needle. The donor/recipient subcutaneous inoculation sequence was carried out twice per recipient per day (1× left neck and 1× right neck) for 5 consecutive days; hence each recipient received a total of 10 inoculations of PBS using a shared needle from donor to recipient.

#### Intradermal recipients

A 1 ml syringe was filled with 1 ml sterile PBS and using a 25 g needle 100 μl of PBS was inoculated via an intradermal route into the skin of the inner thigh of the donor sheep followed by inoculation of 100 μl of PBS into the skin of the inner thigh of the intra-dermal recipient sheep using the same needle. Each intradermal recipient sheep received 5 × 100 μl intradermal inoculations/ day for 5 consecutive days. In total each intradermal recipient received 25 inoculations of PBS by shared needle.

Donor animals were rotated each day to ensure distribution of subcutaneous and intradermal inoculation among donors as well as trying to minimise the effects of possible differences in donor virus replication (Table 2).

Following treatment each sheep was observed regularly for clinical signs of bluetongue disease (BT) and rectal temperature was taken daily for the first 14 days and subsequently on sampling days only until the end of the experiment. Blood samples (EDTA and whole blood) were taken every 1-2 days for the first 10 days and subsequently at 2–5 day intervals until the end of the experiment.

### Cattle

One of the cattle was designated as a “donor” and inoculated subcutaneously with 1 ml BTV-8KC2 into the left side of the neck. The remaining 4 cattle were designated as a “recipient” group. From day 7 post-infection of the donor, subcutaneous inoculations between donor and recipient cattle were carried out as described for the subcutaneous recipient sheep, but for 4 consecutive days only. Each animal of the recipient group received 8 inoculations of PBS in total using a shared needle from donor to recipient cattle.

### BTV detection

#### RNA extraction and real-time RT-PCR (rRT-PCR)

RNA extraction from EDTA blood samples and real-time RT-PCR (rRT-PCR) was carried out as described previously^44^. To control for inter-plate variation between independent extractions and rRT-PCR, several replicates (for a 96 well plate a minimum of 5) of BTV RNA positive sheep blood with a known C_q_ value were included on each extraction/rRT-PCR plate and the sample results of respective plates were only accepted if the positive control samples were within +/− 2 C_q_ values of the previously determined value. Uninfected ruminant EDTA blood samples were included as negative controls.

#### Serological analysis

Whole blood samples were centrifuged at ~2000 *g* for 5 min and 1 ml of serum was transferred to sterile microfuge tubes and stored at +4 °C. Selected serum samples were screened for anti-BTV antibodies using the Early BTV detection kit (ID Vet France) according to the manufacturer’s protocol. The presence of anti-BTV antibodies in samples was determined as % sample/positive control with samples >30% S/P categorised as positive.

#### Virus titration

EDTA blood samples were washed three times using Ca/Mg-free PBS and sonicated for a few seconds at 3000 Hz until red blood cells were lysed. Serially diluted samples were added to freshly passaged KC monolayers in 96-well plates, incubated for 10–14 days at 25 °C followed by testing of the supernatants using an indirect sandwich antigen ELISA as previously described^45^.

#### Modelling approach to determine mechanical transmission frequency

The rRT-PCR data was used to estimate the probability of transmission for each challenge and the duration of the incubation period (defined as time from inoculation to the first detection of BTV RNA) following successful transmission. A mathematical model was constructed which explicitly acknowledged that each animal could be in one of three states at the time of testing: (1) infected and having completed the incubation period; (2) infected but yet to complete the incubation period, or (3) uninfected. The probability of an animal being in each of these states depended on its challenge history, the probability of transmission per challenge and the duration of the incubation period following successful challenge.

Within the study each animal received several potentially infectious challenges by sharing needles between donor and recipient animals multiple times. Therefore, a model was chosen that estimated the probability of transmission per challenge (i.e. potential needle-sharing transmission event) rather than for each individual animal. The duration of the incubation period was assumed to follow a gamma distribution (with mean *θ* and shape parameter *k,* meaning that the variance of the distribution is *θ*^2^/*k*). Parameters were estimated in a Bayesian framework to allow for the incorporation of prior information about the expected duration of the incubation period, based on previous experimental infection studies with this BTV strain^36^.

For each set of experiments, to determine whether the probability of transmission was affected by the level of RNAemia of the donor animal (measure by rRT-PCR), a second model was also fitted in which the probability of transmission depended on the C_q_ value of the blood taken from the donor at the time of attempted transmission. A full description of the model and mathematical equations is given in Supplementary Information Methods online.

## Additional Information

**How to cite this article**: Darpel, K. E. *et al.* Using shared needles for subcutaneous inoculation can transmit bluetongue virus mechanically between ruminant hosts. *Sci. Rep.* **6**, 20627; doi: 10.1038/srep20627 (2016).

## Supplementary Material

Supplementary Information

## Figures and Tables

**Table 1:** Viral titres detected in the blood of BTV-8 infected donor animals on days post infection indicating the highest load of viral RNA as determined by rRT-PCR.

Donor ID	Day p.i./ day of needle sharing	C_q_ -value	Virus titre on KC cells/ TCID_50_/ml
Sheep D1	D5 p.i./D1	23.57	10^5^
	D6 p.i./D2	23.18	10^5^
Sheep D2	D5 p.i./D1	20.31	10^6^
	D6 p.i./D2	19.11	10^6.25^
Sheep D3	D5 p.i./D1	25	10^5.25^
	D6 p.i./D2	23.95	10^4.5^
Cattle D	D8 p.i./D2	21.48	10^4.75^
	D10 p.i./D4	22.26	10^5.5^

**Table 2:** Donor and recipient sheep combinations for subcutaneous and intradermal inoculation via shared needles.

	Recipient animal numbers				
	Day 1	Day 2	Day 3	Day 4	Day 5
Donor 1	SC-1	ID-2	SC-4	SC-1	ID-2
	SC-2	ID-3	ID-1	SC-2	ID-3
	SC-3			SC-3	
Donor 2	SC-4	SC-1	ID-2	SC-4	SC-1
	ID-1	SC-2	ID-3	ID-1	SC-2
		SC-3			SC-3
Donor 3	ID-2	SC-4	SC-1	ID-2	SC-4
	ID-3	ID-1	SC-2	ID-3	ID-1
			SC-3		
