# Supplementary material for: Using shared needles for subcutaneous inoculation can transmit bluetongue virus mechanically between ruminant hosts
Source: Sci Rep. 2016 Feb 8;6:20627. doi: 10.1038/srep20627 (PMC4745043; doi:10.1038/srep20627)
Supplement: Supplementary Information [file srep20627-s1.pdf]

Supplementary Information Methods

Title: Using shared needles for subcutaneous inoculation can transmit bluetongue virus mechanically between ruminant hosts

Authors:

Karin E. Darpel[1,2,*], James Barber[1], Andrew Hope[1, 3], Anthony J. Wilson[1], Simon Gubbins[1], Mark Henstock[1], Lorraine Frost[1], Carrie Batten[1], Eva Veronesi[1,4], Katy Moffat[1], Simon Carpenter[1], Chris Oura[1,5], Philip S. Mellor[1], Peter P.C. Mertens[1]

Modelling approach

To investigate the relationship between C_T value and the probability of transmission two models were considered. In the first model, the probability of transmission ($p(t)$) at time t is related to the C_T value ($C(t)$) as follows,

$$p(t) = 1 - \exp\left(-\exp\left(\alpha + \beta C(t)\right)\right), \quad (0)$$

where α and β are parameters. In the second model, the probability of transmission is assumed to be independent of C_T value, so that,

$$p(t) = 1 - \exp\left(-\exp\left(\alpha\right)\right), \quad (0)$$

where α is a parameter (cf. equation (0) with β=0).

The duration of the period between infection and the appearance of a detectable result via qPCR (equivalent to the incubation period) was assumed to follow a gamma distribution with shape parameter k and mean θ (so that the variance of the distribution is θ^2/k). The times of

the last negative qPCR result and the first positive qPCR result were used to define the interval during which the incubation period was completed.

Parameter estimation

Parameters were estimated in a Bayesian framework. The likelihood for the data needs to account for the possibilities that: (i) those animals which generated a positive qPCR result could have been infected by any of the inoculations (and we do not know which); and (ii) those animals which did not generate a positive qPCR result may not have been infected or were infected, but had yet to complete the incubation period before the end of the experiment. Furthermore, we assume that each challenge acts independently and multiple successful challenges do not result in a shorter incubation period.

For an animal which generated a qPCR result, its contribution to the likelihood is given by,

$$L_{POS}(\alpha, \beta, k, \theta \mid t_{neg}, t_{pos}) = \sum_{j=1}^{J} \left\{ p(t_j \mid \alpha, \beta) \prod_{i=1}^{j-1} \left(1 - p(t_i \mid \alpha, \beta)\right) \right\} \int_{t_{neg}-t_j}^{t_{pos}-t_j} f(t \mid k, \theta)\, \mathrm{d}t, \quad (0)$$

where J is the total number of challenges, t_j is the time of the jth challenge, t_{neg} is the time of the last negative qPCR results and t_{pos} is the time of the first positive qPCR result. The first term in the summation (in braces) in equation (0) is the probability that the animal became infected on the jth challenge (and was not infected in any of the previous challenges) and the second term is the probability that the animal would complete its incubation period between the times of the last negative and first positive qPCR result.

For an animal which did not generate a positive qPCR result, its contribution to the likelihood is given by,

$$L_{NEG}(\alpha,\beta,k,\theta \mid t_{end}) = \sum_{j=1}^{J}\left\{p(t_j \mid \alpha,\beta)\prod_{i=1}^{j-1}\left(1-p(t_i \mid \alpha,\beta)\right)\right\}\int_{t_{end}-t_j}^{\infty} f(t \mid k,\theta)\,\mathrm{d}t + \prod_{j=1}^{J}\left(1-p(t_j \mid \alpha,\beta)\right) \qquad (0)$$

where t_{end} is the time at which the experiment ended and the remaining variables and parameters are the same as in equation (0). The first term in equation (0) is the probability that the animal was infected on the jth challenge but had yet to complete the incubation period by the end of the experiment (cf. equation (0)), while the second term is the probability that the animal did not become infected.

Finally, the likelihood for the complete data set is given by,

$$L(\alpha,\beta,k,\theta) = \prod_{a=1}^{A} L_{POS}(\alpha,\beta,k,\theta \mid t_{neg}^{(a)},t_{pos}^{(a)})^{\delta_a} \times L_{NEG}(\alpha,\beta,k,\theta \mid t_{end})^{1-\delta_a}, \qquad (0)$$

where a identifies the animal (a=1,2,…,A), δ_a is a variable indicating whether (δ_a=1) or not (δ_a=0) animal a generated a positive qPCR result and L_{POS} and L_{NEG} are the individual contributions to the likelihood defined by equations (0) and (0), respectively.

Non-informative priors (diffuse Normal with mean zero and standard deviation 100) were used for the parameters in the probability of transmission (i.e. α and β in equation (0) or (0)). Informative priors for the incubation period parameters (k and θ) were calculated by fitting a gamma distribution to the incubation periods reported in [1]. All priors were assumed to be independent of one another.

Samples from the joint posterior distribution for each model were generated using an adaptive Metropolis algorithm [2] in which the scaling factor for the proposal distribution was tuned during burn-in to ensure an acceptance rate of between 20% and 40% for efficient

sampling of the target distribution [3]. Three chains were run for 500,000 iterations after burn-in, with an appropriate burn-in selected based on assessment of chain convergence (both visually and by the Geweke diagnostic method [4]).

The two models, (0) and (0), were compared for each experiment (i.e. cattle, sheep intradermal and sheep subcutaneous), so that a total of six models were fitted to the experimental data. If the 95% CI for b included zero, we concluded that there was insufficient evidence to infer an effect of donor PCRemia on the probability of transmission and rejected model (1) in favour of model (2).

References

1 Gubbins, S., Hartemink, N. A., Wilson, A. J., Moulin, V., Noordegraaf, C. A. V., van der Sluijs, M. T. W. *et al.* Scaling from challenge experiments to the field: Quantifying the impact of vaccination on the transmission of bluetongue virus serotype 8. *Preventive Veterinary Medicine* **105**, 297-308, doi:10.1016/j.prevetmed.2012.02.016 (2012).

2 Haario, H., Saksman, E. & Tamminen, J. An adaptive Metropolis algorithm. *Bernoulli* **7**, 223-242 (2001).

3 Andrieu, C. & Thomas, J. A tutorial on adaptive MCMC. *Statistics and Computing* **18**, 343-373 (2008).

4 Plummer, M., Best, N., Cowles, K. & Vines, K. CODA: Convergence Diagnosis and Output Analysis for MCMC. *R News* **6**, 7-11 (2006).
